# Rates, risk factors & methods of self harm among minority ethnic groups in the UK: a systematic review

**DOI:** 10.1186/1471-2458-7-336

**Published:** 2007-11-19

**Authors:** Kamaldeep Bhui, Kwame McKenzie, Farhat Rasul

**Affiliations:** 1 Centre for Psychiatry, Barts and The London, Queen Mary's School of Medicine and Dentistry, Old Anatomy Building, Charterhouse Square, London EC1M 6BQ, UK; 2 East London & City Mental Health Trust, London, UK; 3 Centre for Addictions and Mental Health, Toronto University, Toronto, Canada; 4 University College London, London, UK

## Abstract

**Background:**

Studies suggest that the rates of self harm vary by ethnic group, but the evidence for variation in risk factors has not been synthesised to inform preventive initiatives.

**Methods:**

We undertook a systematic literature review of research about self harm that compared at least two ethnic groups in the United Kingdom.

**Results:**

25 publications from 1765 titles and abstracts met our inclusion criteria. There was higher rate of self harm among South Asian women, compared with South Asian men and White women. In a pooled estimate from two studies, compared to their white counterparts, Asian women were more likely to self harm (Relative Risk 1.4, 95%CI: 1.1 to 1.8, p = 0.005), and Asian men were less likely to self harm (RR 0.5, 95% CI: 0.4 to 0.7, p < 0.001). Some studies concluded that South Asian adults self-harm impulsively in response to life events rather than in association with a psychiatric illness. Studies of adolescents showed similar methods of self harm and interpersonal disputes with parents and friends across ethnic groups. There were few studies of people of Caribbean, African and other minority ethnic groups, few studies took a population based and prospective design and few investigated self harm among prisoners, asylum seekers and refugees.

**Conclusion:**

This review finds some ethnic differences in the nature and presentation of self harm. This argues for ethnic specific preventive actions. However, the literature does not comprehensively cover the UK's diverse ethnic groups.

## Background

Non-fatal self harm (SH) is common [1]. It places significant burden on care services. The degree of suicidal intent is often difficult to assess, but SH is the most important risk factor for later completed suicide [2,3] and future self-harm [4]. Mental distress and illness are both risk factors for suicide and self harm. Though there are many studies that show ethnic variations in prevalence of mental illness, and access to care and treatment [5], few have considered the cultural epidemiology of self harm.

Cross cultural investigations of self harm and suicide are necessary to inform future preventive initiatives if these are to be effective across diverse ethnic groups [6,7]. Cultural influences are proposed to explain some of the ethnic variations in rates and methods of self harm within any one country and across countries [8-10]. In addition, factors known to increase the risk of mental distress, social exclusion, unemployment, and financial strain are more common in some ethnic groups and may increase the risk of self harm among these ethnic groups [11,12].

Clinical assessment and preventive policies rarely take into consideration potentially distinct needs of specific ethnic groups. This partly reflects that the evidence base is not accessible or synthesised to guide practitioners or policy makers to consider ethnic differences in rates and risk factors. This paper reports the findings of a systematic review of UK studies investigating self harm amongst at least two minority ethnic groups. The aim of the review was to establish whether there were ethnic differences in prevalence rates, clinical presentations including risk factors, and methods of self harm across the largest minority ethnic groups in the UK. We hypothesised that prevalence, risk and methods of self harm would differ between South Asian, Black Caribbean, and White British people.

## Methods

**Figure 1:**
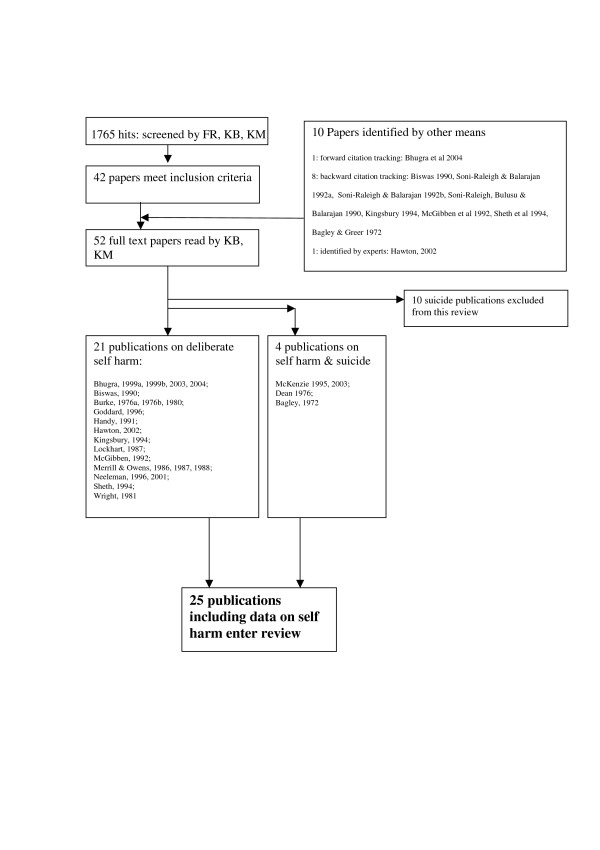
Flow chart of identified papers.

The literature review is part of a larger mixed-methods study investigating suicide and self harm in minority ethnic groups commissioned by the National Institute of Mental Health England and the Department of Health's 'Delivering Race Equality' strategy. The paper presents a review of self harm only, but because many studies investigate self harm and suicide, our search strategy targetted both publications on suicide and self harm. Specifically, we searched for publications that included data on a) self harm or suicide, b) in at least two ethnic groups living in the UK, and c) that were published in English. Titles and abstracts of these studies were screened (FR, KB, KM). Full text papers were obtained if from the abstracts the studies were confirmed to a) compare rates or clinical risk factors or methods of self harm or suicide; b) include data on 2 or more ethnic groups living in the UK; c) studies were published in English between 1960 and the start date of the project, 2004. Where there was uncertainty, the full text paper was retrieved. The following databases: MEDLINE, PSYCHINFO, EMBASE and CINAHL. The search strategy used the following search terms: SUICIDE or SUICIDAL IDEATION, or SUICIDAL THOUGHTS, or SUICIDE BEHAVIOUR or SELF-HARM or DELIBERATE SELF-HARM. The strategy was devised with the assistance of a librarian familiar with the databases and MESH terms needed to optimise the search. The findings from this search were combined with a search on ETHNICITY or ETHNIC or RACE. There were 1765 hits. In this paper we review the 25 studies that met our inclusion criteria (see Figure 1).

Two reviewers (KB KM) independently assessed the methodological quality of the studies using a scoring scheme adapted from previous reviews (adapted from [5]; Table 1). Data were extracted and tabulated independently by the two reviewers who tabulated study characteristics (Table 2), clinical risk factors for self harm and methods of self harm (Additional file 1) and prevalence or rates of self harm (Table 3). Adolescent studies were separately reported given the distinct risk of mental health problems, service use and developmental challenges for adolescents.

**Table 1:** Paper quality scoring system on five criteria:

1	Size of the study population of relevance	Range 0–1
2	Power calculation: whether performed or not	Range 0–1
3	Confounding: degree of adjustment for obvious confounders	Range 0, none; 1, age/gender; 2, age/gender and socio-economic indicators
4	Ethnicity definition: whether explicit, accurate and hypothesis based	Range 0, none; 1, a definition; 2, self assigned; 3 rated on basis of self identification and parental origins
5	Deliberate self harm: whether attempts had been made to ensure the episode was an actual self harm	Range 0, none; 1 self report; 2, some attempt at measurement; 3, well defined and measured

The maximum possible score was 10. This schema was piloted to ensure that it discriminated adequately between the papers included. How well a study measured deliberate self harm and how accurately it measured ethnicity were weighted more highly than other criteria because they were considered particularly important for this review

**Table 2:** Characteristics of studies on self harm in ethnic groups in the UK

Author	Ethnicity definition	Population from sample is taken	Sample size	DSH or Suicide rating scale?	Data sources	Quality Rating
*Bagley 1972	Defined by oppression. Black refers to those who lack economic power, and do not control their destinies. White as oppressors.	South London A&E Department. 206 consecutive Admissions to casualty Followed up over 18 months	25 Black (2 completed suicide, 23 recovered) Black included the following: Caribbean (48%), Indian and Pakistani (20%), African (16%) Cypriot (16%). 25 White in comparison group	No	Hospital records	2
*Bhugra 2003	South Asian (Indian sub-continent origin) or White	All adolescents presenting to local East London Hospital in 1 year period for DSH	76 cases: 15 South Asian (12 women) 46 White (38 women) 6 Middle Eastern (4 women) 9 Black (9 women)	No	Case notes and interviews	1
*Bhugra 2004	South Asian: self ascribed origin: individuals whose parents, grandparents or themselves originate in Indian sub-continent	All adolescents presenting to hospital services in West London in a 1 year period for DSH	76 cases: 15 South Asian (12 women) 46 White (38 women) 6 Middle Eastern (4 women) 9 Black (9 women)	No DSH scale DACS (DSH Assessment by Clinicians Schedule)	Case notes and interviews	2
*Bhugra 1999a	Self-ascribed OPCS census categories & case notes.	Patient's aged 16–64 presenting with DSH to general, medical, accident or psychiatric services within local hospital in West London.	434 patients presenting with suicidal behaviour. 90 Asian cases of attempted suicide	No Numbers of people presenting with DSH or attempted suicide	Hospital records, interview, GP records	4
*Bhugra et al 1999b	Self-ascribed OPCS census categories & case notes.	Patients aged 16–64 and resident in the areas for at least six months, presenting with DSH to general, medical, accident or psychiatric services in West London	Comparison of first 27 Asian women presenting with DSH with 27 matched controls (Asian women attending GP surgeries for other reasons)	Numbers of DSH or attempted suicide (DACS : DSH Assessment by Clinicians Schedule))	Hospital records, interview	4
* Burke 1976a	Self assigned Place of birth known for 2695 cases Of 2075 with no place of birth, a 10% sample selected and case notes examined. 2.5% were of Asian origin	Admissions following attempted suicide to Birmingham hospital during a four year period: 1969–1972 Under 65s admitted with poisoning	68 admissions with Asian place of birth 16 cases: iatrogenic or accidental cases 52 deliberate self poisoning cases	No. Admission to Birmingham hospitals following self poisoning used as entry criterion and definition for inclusion	Case notes	3
*Burke 1976b	Self assigned Place of birth known for 2695 cases Of 2075 with no place of birth, a 10% sample selected and case notes examined. 2.5% were of Asian origin	Admissions following attempted suicide to Birmingham hospital during a four year period: 1969–1972 Under 65s admitted with poisoning	60 admissions with place of birth in West Indies	No	Case notes	3
* ≠ Burke 1980	As Burke 1976 a, b-same samples	Attempted suicide (1969–1972) and admitted to Birmingham hospitals 100 patients followed up until Jan 1969-average follow up period of 5 years	52 West Indian (3 had made 2 previous attempts) 48 Asian (three had made two attempts and one died at admission)	No Classification based on attempted suicide and psychiatric history: 1. no psych history, 2. psych care at time of attempt 3. psych care before attempt 4 psych care following attempt	Medical, psychiatric records from GP, hospital and coroner	3
*Biswas 1990	Not given Asians selected on basis of Asian names	A & E presentations to Bradford Royal Infirmary, with self poisoning before or on 1^st ^March 1987	72 (38 Asian, 34 White) adolescents aged < or = 17 yrs	No "self-poisoning" from case notes	Case notes	1
*± Dean et al 1976	Place of birth: country comparisons of rates of Non-fatal self poisoning: but no comparison of ethnic groups.	All deaths registered as suicides between 1970–72	333	Admissions for poisoning and adverse reactions in E, W & § Rep of Ireland Assumed all for DSH and not adverse reactions: assessed assumption on 80 admissions. Of 80, 82.5% confirmed to be DSH.	Population census	5
*Goddard 1996	Based upon 1991 census. GP notes for clarification on 22 of them: 14 white, 7 black, 1 other. Black Caribbean, Black African, Black Other, Indian, Pakistani, Bangladeshi, Chinese, Asian, Other White. Groups collapsed into white, black, and other Asian	Total referrals to specialist centre at Maudsley Hospital Age > 10 First referral following DSH	Total 100 White: 64 Black :28 Other 8	No Standardised Maudsley assessment sheet: summary of symptoms, diagnosis, treatment, emotional symptoms and antisocial behaviour	Hospital records and OPCS	2
*Handy 1991	Parent & children's place of birth Parents UK born = British Asian born = Asian	Presenters to child guidance clinic, Walsgrave hospital Coventry	50 Adolescents 25 Asian 25 White	No Self poisoning on presentation	Medical records	1
± Hawton 2002	None given White Asian Black Other	Pupils in 41 schools in England: Oxfordshire, Northamptonshire, and Birmingham. 90% of pupils aged 15 and 16	6020 pupils White 2356 Asian 371 Black 68 Other 74	Question on self harm Asked for description of the act and consequences Classified by 3 independent ratings using agreed definitions	Self report questionnaire	6
*Kingsbury 1994	No definition Asian: Indian, Pakistani, Sri Lankan	12–18 year old attendees at West Middlesex hospital A&E. following overdose, Feb 1987-April1998	50 Total 113 Asian, 37 Caucasian 13 lost due to premature discharge and failure to attend follow up OPA	Pallis suicide intent scale	Hospital records, semi-structured interview questionnaire	4
*Lockhart 1987	Ethnic origin: no definition Asian West Indian, Europid	Adults admitted after self poisoning to inner London General hospital. Two time periods: from 1) Nov 1971 to Feb 1972. 2) Sept 1983-June1984 Adults over 15	1971/72 Europid = 80 Asian = 1 WI = 0 Other = 1 1983/84 Euro = 72 Asian = 6 WI = 7 Other = 1	No	Questionnaire completed for each patient	0
*McGibben 1992	No definitions Denominators established from Asian names in electoral register and racial breakdown obtained from school data	Admissions to Coventry hospitals for overdose aged < 16 between 1982–90 92% of admissions referred for opinion and therefore included in sample	340 referrals for overdose 1982–90 295 admitted for DSH 222 girls 73 boys 45 Asian of which 37 were girls	No. Deliberate self poisoning	Hospital records, population denominators from city of Coventry, 1981 census data, Coventry school pupil census	0
*McKenzie 1995	White i.e. self or parents UK born, Afro-Caribbean =both parents born in Caribbean	Admissions for psychosis to 2 London psychiatric hospitals 1985/March 986 – Feb 1988 Oct 1988-August 1989 Recent (5 year) onset of psychosis Four year follow up	191 in total, but follow up data on 166 (87%) available 53 Afro-Caribbean 60 White	No WHO life chart HADS WHO disability assessment schedule PSE at baseline	Interview	7
≠ McKenzie 2003	Observer defined OPCS 1991 and Gen Register for Scotland White = OPCS white with mother and father born in the UK Caribbean = OPCS Black-Caribbean or Black Other with mother or father born in Caribbean or UK	People with psychosis in a case management trial 3 London 1 Manchester centre	708 Baseline 203 Caribbean 234 White British Follow up attrition AC: 26 (13%) WB: 35 (15%) Drop outs: AC 20 & 25 WB	No. WHO Life Chart: asked if attempted suicide and frequency Rated on patient report and all sources of information including case notes/relatives/managers	Case notes, interview with family, client, case managers	8
*Merrill 1986	Whites: born in England, Scotland & Wales South Asians: born in India. Pakistan, Bangladesh, UK (n = 52, 26.5%) & East Africa (5.26%)	Admission to West Midlands Poisons unit for self poisoning with mediation or taking substances not fit for consumption 1 Jan 1979 to 31 Dec 1981	1171 975 White 196 Asians (59% Indian, Sikh)	No	Admissions records. Coding sheet completed by admitting clinicians	4
*Merrill 1987	Place of birth	Admission to West Midlands Poisons unit for self poisoning with mediation or taking substances not fit for consumption 1 Jan 1979 to 31 Dec 1981	975 White 130 West Indian (55 born in WI, 75 born in UK but WI parentage)	No	Admissions records. Coding sheet completed by admitting clinicians	2
*Merrill 1988	Place of birth Irish Scottish English Indian/Pakistan West Indian	A & E attendees at West Birmingham. DSH leading to admission to West Midlands poisons unit Birmingham 1^st ^Jan 1979 to 31^st ^Dec 1981	467 Asian: 36 West India: 16 Scottish: 17 English: 364 Irish: 34	No	Admissions records. Coding sheet completed by admitting clinicians	3
*Neeleman 1996	Region of birth, ethnicity from OPCS 1991 census: White Black Caribbean Black African Other, Indian Pakistani Bangladeshi Chinese Asian Others	A&E attendees at Kings College Hospital, for intentional overdose, over 6 month period in 1991: 237 patients attended DSH team assessed 48% (113/247) of all attenders.	Total 105 Ethnic Group 83 White (78) 11 African Caribbean (4) 7 Asian (1) 4 African (2)	No Attendance and psychiatric assessment were criteria	Hospital records OPCS	3
*Neeleman 2001	Observer assigned OPCS categories collapsed into: White African Caribbean Asian	A & E attendees with DSH at two London Hospitals: King's College Hospital (KCH) and Lewisham Hospital (LH).	Attenders: 2352 Analyses for 1643 attending own A&E. Unknown ethnic origin: 184 KCH LH 1341+1011 White: 720 + 608 AC : 212 + 71 Asian : 9 + 23	No Attendence for DSH, excluded accidents and uncertain cases	Hospital records. 1991 census	6
*Sheth 1994	None	Adult admitted to Yorkshire burns unit, March 1983 to March 1993, with suicidal burns injury	234 admissions, 20 of which had suicidal burns injury, 17 of whom were women Of the women 14 (70%) were Asian	No	Case notes	1
*Wright 1981	Asian (from the Indian sub continent-majority Sikhs) West Indian Caucasian	Admissions to Dudley Road Hospital 1976–79	2001 Total 1665 White 160 Caribbean 176 Asian	No	Questionnaire on social, physical, psychiatric history. Filled in by doctors	0

*Cross sectional studies, usually a case series using ethnic group comparisons, and associations between those with and without self harm.

≠ Prospective.

± Population based study using schools as sampling frame

**Table 3:** Rates of Self Harm across Ethnic groups in the UK

**Studies of Adults**												
	Units of Rates	Population of Interest	Statistics as reported in the paper									Summary of Main Findings

Bhugra et al 1999a	Attempted suicide Per 10 000 person years	Women		Women	Men							Young South Asian women are vulnerable to increased rates of DSH.
				Rate, 95%CI	Rate, 95%CI							Attempted suicide rates highest in South Asian women than other ethnic groups but highest in white men, maybe because inclusion of Irish in white category.
			South Asian	37.7, 29.02–47.1	13.9, 8.9–20.8							In men highest rate in Whites aged 16–24 and Black people aged 25–34.
			White	23.3, 19.7–27.4	24.6, 20.9–28.7							
			Black	23.9, 13.9–38.7	11.3, 4.6–23.2							
			Other	30.1, 17.6–48.2	8.7, 2.8–20.3							
			Attempted suicide rates highest in South Asian women aged 16–24 (92.7/10000 person years), aged 25–34 (34.4) and also high in "other" ethnic women aged 16–24 (75.9 per 100000 person years) and aged 25–34 (41.1).									
			White men also had high rates compared with other ethnic groups in 16–24 (30.2), 25–34 (28.1), 35–44 (30.1), as did black men in 25–34 age group (31.8).									
Burke 1976a	Per 100 000 population	South Asian Adults	Age	Female Rate	Male Rate	Total Rate						Proportion of South Asians in self harm group was 60% of those expected considering population distributions
			15–24	213	74	123						
			25–44	95	70	66						
			45–64	32	0	9						
				216	57	79						
			Rate lower than native population (57/100,000, 126/100,000).									Women more common among 15–24 group and men more common at later ages
Burke 1976b	Per 100 000 persons years	West Indian Adults	Age	Female	Male	Total						Rates among Caribbean females aged 15–24 twice that of males same age.
			15–24	514	101	336						
			25–44	84	66	74						
			Total	180	56	113						Low risk of attempted suicide in Caribbean people
Dean et al 1976	Rates	Adults	Rates increasing in all countries, especially in 15–44 age groups, especially among women.									Country comparison of rates, England & Wales rate is greater than those of Scotland, Northern Ireland, or Republic of Ireland
			Rates among women in England and Wales are twice the rate for Scotland and Ireland, and four times the rate for Republic of Ireland.									
Lockhart et al 1987	Risk expressed as % at two time points 1971 and 1984	Adults	1971: 93 patients admitted on 100 occasions from population of 92 720									An increase in admissions for among West Indians for self-poisoning.
			1983/84: 86 patients admitted on 94 occasions from 73 929									
			Risk of admissions per week halved from 5.8 to 2.5									
			Incidence rate halved from 326 to 178/100 000 persons a year									
			Rise in WI admissions from 0 to 7%, p < 0.05, fall among South Asians: 1 to 7%. P = 0.13, fall of Europids, p < 0.01									
			In 1971 and 1981, 6.4% of local population of WI origin									
			In 1983/4: 7% of local population of WI origin									
McKenzie et al 2003	OR: unadjusted and adjusted for age, gender, MADRS, education, Diagnosis, time since onset)	Adults		AC	WB	OR	OR (adjusted)					Lower prevalence of suicidal behaviour in Caribbean origin people with psychosis not present in under 35's.
			Attempted Suicide (AS)	14	29	0.52, 0.26 to 1.02	0.54, 0.26 to 1.13					
			AS for < 35 yo	12(13%)	13 (18%)	0.7, 0.36 to 1.51	0.93, 0.37 to 2.32					
			AS for > 35 yo	2(2.1%)	16 (11.4%)	0.17 (0.04–0.71)	0.19 (0.04 to 0.89)					
			AS or Completed Suicide	17	33	0.56, 0.3 to 1.03	0.59, 0.3 to 1.14					
			(age/gender adjusted = 0.49, 0.26 -0.92, p = 0.06)									
			Caribbean origin patients aged > 35 5 times less likely to attempt suicide									
			No difference in under 35's.									
			Attempted & completed suicide combined Caribbean origin patients aged > 35 4 times less likely.									
			Caribbean origin patients aged < 35 risk same as British whites									
Merrill et al 1986	Rates of self poisoning per 100 000 per year	Adults			UK born	Asian born	p value					Rate for self-poisoning higher in Asian females than white females. Culture conflict important. Asian patients less likely to have previously self-poisoned, received psychiatric treatment or psychiatric diagnosis.
			Men	25–34	438	146	< 0.0005					
				35–44	240	80	< 0.025					
				> 44	98	28	< 0.05					
				Overall	190	102	< 0.0005					
			Women									
				Overall	299	376	< 0.05					
Merrill et al 1987	Rates of self poisoning/100 000 per year	Adult			West Indian	White British	p					Not very different from Asians, could be a reflection of service characteristics rather than ethnic group.
			Overall	Men	103	190	< 0.025					
				Men 25–34	129	438	< 0.05					
				Women > 45	30	133	< 0.05					
			Rates for over 25s lower for men WI (men: p < 0.001; women: p < 0.005)									
Merrill et al 1988	Rates per 100 000 per year	Adult	Men	Asians	West Indian	English	Irish	Scottish				Asian aribbean young females had greater rates than English females. Irish & Scottish of both sexes had higher rates than English.
			25–34 yo:	146*****	129	406	662	1199*				Power and denominator problems
			> 35	48**	57*	131	156	462*****				
			< 16	113*****	98***	236	243	648*****				
			Women									
			25–34 yo:	501	257	447	853*	565****				
			> 35	90	37	167	367*****	547****				
			< 16	402	229*	341	456*	626*				
			*p < 0.05, ** p < 0.01 ***p < 0.005 ****p < 0.001 *****p < 0.0005									
Neeleman et al 1996	Referral Ratios	Adult	Unadjusted Referral Ratios (Ethnic groups generally younger) for all subjects									Indian female rates of Self Harm are 2.6 times the rate of white women
				Men	Women	All						UK born Indian females had 7.8 times rate of UK born white females. Unemployment associated with a 9-fold increase in referral rates in whites and 3-fold increase in minorities.
			Black Caribbean	0.56, 0.16–1.43	0.61, 0.24–1.26	0.61, 0.3–1.09						
			South Asian	0.39, 0.01–2.17	1.68, 0.61–3.66	1.15,0.46–2.37						
			Indian	0	2.6, 0.53–7.6							
			All Ethnic groups	0.45, 0.17–0.98	0.78, 0.45–1.27	0.65, 0.41–0.99						
			Unadjusted Referral Ratios (Ethnic groups generally younger) for UK born only									
				Men	Women	All						
			AC	0.19, 0.00–1.05	0.41, 0.08–1.20	0.32, 0.09–0.82						
			AS	1.2, 0.03–6.68	3.53, 0.96–9.03	2.55, 0.83–5.95						
			IN	0	7.76, 1.6–22.66							
			Cross validation study: ethnicity assigned on basis of name, to check that referral to DSH may be biased by ethnic group rather than reflect attendance. Only possible for Indian names									
			Attendance ratio Indian Men (indirect standardization): 1.92, 0.52–10.24; Indian women: 3.07, 1.4 to 5.8									
Wright 1981	Annual increase in % admissions	Adult	Mean annual increase %		% born in country of origin for ethnic group							Self-poisoning is an inarticulate cry for help or even a cry of frustration.
					1976	1979						
			Caucasian	9.5	100	100						
			West Indian	31.5	69.6	48						
			Asian	14.5	83.9	70.3						

**Studies of Adolescents**												

Hawton 2002	Prevalence	Adolescents	Males					Females				Lower risk among South Asian females not sustained in multivariate analyses
				No	% self harm	OR	95%CI	No	% self harm	OR	95%CI	
			White	2536	3.3	1		2727	11.6	1		
			Asian	371	2.7	0.82	0.42–1.58	254	6.7	0.55	0.33–0.91	
			Black	68	0	-	-	89	6.7	0.55	0.24–1.27	
			Other	74	6.8	2.14	0.84–5.85	72	13.9	1.23	0.62–2.42	
McGibben et al 1992	Per 1000 persons a year	Adolescents	Overall rates: 2.33/1000/year									Excess admission for DSP in Asian and white girls
			Asians = 2.47/1000/year									
			White = 2.31/1000/year									
			Asian boys: 0..88/1000/year	White Boys 1.14/1000/year								
			Asian girl: 4.06/1000/year	White Girls: 3.47/1000/year								
			(NS)									
			Religion: NS, but a trend for more Sikhs to be admitted: Sikh 3.17/1000/year, Muslim 1.76, Hindu 1.46									
Bhugra et al 2003	Rates of DSH per 10 000	Adolescents		White	South Asian							Female Self Harm rates are greater than male rates for South Asians & Whites.
			Male aged 10–14:	7.3	9							Rates for South Asian and White women aged 14–15 do not differ much
			Male aged 14–15:	62	-							Self Harm rates greater in white females aged 10–14 compared with South Asian
			Female aged 10–14:	51	21							
			Female aged 14–15:	158	127							
			Female aged > 15:	44	-							

The largest ethnic minority groups living in the UK are migrants and their descendents from the Indian sub-continent, often called South Asians in the UK. Other migrants and their descendents are from the Caribbean and Africa; these ethnic groups are often called Black Caribbean or African Caribbean if of Caribbean origin and Black African if of African origin. These categories are those used in the 2001 census in the UK. As much as possible we have tried to group different studies together by ethnic minority groups used in the 2001 UK census; but we have retained the group names used by the authors of studies in the tabulated data.

## Results

### Methodological Quality

Sixteen studies obtained a low score (3 or less) on our quality assessment. Seven scored between 4 and 6 (medium score), and two studies scored over 7. Most of the studies scored less than five on quality as they had broad definitions of ethnicity, and adjusted only for age and sex [e.g. [13-15]], or only presented rates of deliberate self harm and poisoning without a detailed assessment of risk factors across ethnic groups [16-22]. Six of the studies recruited adolescents [16,18,19,21,23,24]; two of these scored between 4 and 6 [19,21].

Seventeen studies adopted ethnic group comparisons using a cross-sectional case series. There was one prospective study of people who had a severe mental illness; they were followed up over four years to assess baseline predictors for future outcomes. There were two population studies [19,25]. Three studies used regression analyses, adjusting for confounders [24,26,27]; one study used age standardization [28], otherwise most studies used age/gender stratification to report rates or presented descriptive data. Half of the studies had less than 30 subjects in all the groups that were compared. Fifteen studies used case notes and other routine sources of data, with the remaining 9 including some interview data, with one using a self report questionnaire [19]. Only four studies used specific instruments to confirm self-harm [17,21,27,29], whereas most studies relied on 'reasons for admission or contact with services' as recorded in the case notes or reported at interview. Hawton et al took special precautions and used three raters to independently validate self harm against an agreed definition [19]. Seven studies included a rate calculation [12-14,17,20,24,30].

Comparison of studies was complicated by the use of different ethnic identifiers. Some studies used self-assigned ethnicity [e.g. [31]], others observer assigned ethnicity and others used place of birth. Ethnic identifiers have changed over time as have ethnic minority groups. Early studies of South Asian groups in the 1970s would be reporting about a group which was predominantly not born in the UK, whereas currently 50% of the England and Wales South Asian origin population was born in the UK. Early studies, for example those of Burke [12,32,33], used the term "West Indian" which is now not commonly used.

Some studies took care to ascribe ethnic group using self or observer assigned census categories [13,17,27], whereas others based it upon place of birth [12,30,32,33]. Ethnicity was more variously defined in studies using data from medical records and for some studies ethnicity was based on South Asian names [16], place of birth [14,15,18], or country of origin [21,22]. In some of these studies it was unclear how ethnicity was defined [19,20,34]. Some studies used black to indicate a number of ethnic groups, for example, two publications defined black as being "West Indian, African, Asian or Cypriot" origin [14,15]. Furthermore, Bagley and Greer classified people with diverse background from the "coloured commonwealth areas of the Caribbean, Africa, India, Pakistan and Cyprus" as "black" [26].

### Adult Studies

#### Clinical Characteristics and Risk Factors

##### Repeated attempts

The majority of studies reported lower rates of repeat self harm in South Asian and Caribbean origin groups. Previous histories of self harm were less common amongst West Indians and South Asians presenting with self harm [12,14,22,27,32]. Bagley et al showed that "Black" people (representing a mixed group of BME groups) were more likely to make a repeat suicide attempt, compared with a White comparison group, if they had not received psychiatric treatment or social care following a self-harm episode [26].

##### Impulsivity

Two studies concluded that interpersonal disputes precipitated the self harm more frequently among immigrant than white patients, and suggested that situational stress more frequently led to impulsive acts of self harm among the black group [12,26,32]. This suggested an absence of ongoing psychiatric illness, but also that self harm emerged in response to interpersonal distress. South Asian women who self harmed were more likely to express regret than a control group; this again suggests that impulsive acts were more common amongst South Asians [31].

##### Psychiatric Disorder

In most studies psychiatric disorders were assessed using clinical diagnoses rather than standardised or validated assessment scales. Evidence of differences in the rates of psychiatric disorder in self harming patients among different ethnic minority groups is not consistent. No one in a South Asian sample of burns victims had a history of psychiatric disorder [34]. No psychiatric diagnoses were found in 77% of White and South Asian presenters to emergency departments [28]. Bhugra et al reported depression was less common among South Asian self harmers compared with their White British counterparts [31]. Merrill & Owens found that West Indians had a lower risk of psychiatric illness when compared with White British subjects [15]. Conversely, Merrill & Owens reported that psychiatric illness was less common among whites when compared with South Asian subjects [14]. Burke et al's early studies found psychiatric disorders among a third of South Asian presenters and depression among 20 of 22 West Indians admitted following self poisoning [12,32]. When investigating a sample of African Caribbean people with psychosis, McKenzie et al reported a lower risk of self harm over a two year follow up period [27].

##### Culture Conflict

Where social or interpersonal distress was related to culturally specific practices, expectations or beliefs, research studies often concluded that 'cultural conflict' was present. Bhugra et al compared a variety of socio-cultural risk factors for self harm in South Asian and White patients [31]. In this case-control study, Asian women who were suicide attempters were matched on age and sex with Asian controls. Suicide attempters were more likely to have less traditional cultural attitudes, to report recent family arguments and to be in a cross-racial relationship with a white person [31]. Only 30% of the suicide attempters reported that arranged marriages were a good idea, while 80% of the control group held these views. Unfavourable attitudes towards arranged marriage seemed to be associated with self harm, and may reflect adverse experiences of arranged marriage which generated mental distress when faced with an arranged marriage, or less traditional attitudes leading to conflict with people in the person's cultural reference group, with or without a personal experience of arranged marriage. Merrill & Owens concluded that culture conflict was more common amongst South Asian patients, particularly unmarried females [14]. In a study of self poisoning cultural problems were also reported more often in South Asians than whites [22]. There was no endorsement of questions about culture conflict in the white or West Indian group. In contrast, 28% of the Asian group reported that cultural problems were the precipitating cause.

##### Ethnic density

This refers to the concentration of specific or all ethnic groups in any one geographical area, reflecting the extent to which ethnic minorities may be living in an area where other ethnic minorities live [35]. Rates of self harm in ethnic minorities increased with greater ethnic density and after peaking it decreased with further increases in ethnic density [35].

#### Methods of Self Harm

A number of studies compared methods of self harm in different ethnic groups [16,17,20,22,31,34,36,37]. Most of these studies collected data on methods of self harm from medical records and interviews with patients seen in hospital departments. These described the types of ingested medication, and that oral ingestion of tablets was a common method of self harm. However, there were only a few significant findings. Domestic substances, such as bleach, coal gas and medications, were used more often by South Asians than other ethnic groups; among West Indians psychotropic medications were as commonly used as analgesics [12,32]. However, two studies concluded that attempted suicide by self poisoning was less prevalent in West Indian immigrants than white patients [12,32]. Sheth et al reported a specific method for South Asian women admitted to a burns unit; over 70% of patients admitted to a burns unit following a suicide attempt were South Asian women who had used paraffin to set fire to themselves [34].

#### Rates of Self Harm

Two studies reported that South Asian and West Indian men and women had lower rates of self harm when compared with the local general population [12,32]. Burke, in a series of studies, showed that admission rates were lower for attempted suicide in immigrants from Commonwealth countries compared with the local Birmingham population [12,32,33]. Nonetheless, there were rate differences by age and gender. Rates were highest in older male immigrants (aged 25 and over) and younger female immigrants (aged under 25).

Subsequent studies have demonstrated high rates of self harm among South Asian and some Caribbean groups [14,15,30,32,35-37], and specifically an 8 fold higher risk among UK born Indian females [28]. Rates of attempted suicide were highest in South Asian women in West London when compared with other ethnic groups living in the same area [17]. These trends were most evident in women aged between 16 and 24. Merrill and Owens also showed a higher rate of self poisoning among South Asian women compared with White UK born women [14]. In a later paper, this research group reported that the rates of self-harm were higher in Caribbean born females aged less than 35 compared with White people born in the UK, in the same age group and admitted to the same poisons units [15]. However, this finding did not show statistically significant differences between the ethnic groups when distinct age bands were compared.

McKenzie et al did show age differences in risk of attempted suicide in Caribbean origin patients given a diagnosis of a psychotic illness [27]. Those under 35 were four times more likely to attempt suicide than those over 35.

Only data from two studies could be included in meta-analysis; these studies compared rates of self-poisoning and attempted suicide among Asian and White people presenting to services [14,17]. We compared pooled data for men and women separately using a random effects model. Asian women were more likely to self harm compared to White women (pooled Relative Risk 1.4, 95%CI: 1.1 to 1.8, p = 0.005). Asian men were less likely to self harm compared with White men (Relative Risk 0.5, 95% CI: 0.4 to 0.7, p < 0.001).

### Adolescent Studies

#### Clinical Characteristics and Risk Factors

Disciplinary issues or arguments with parents were common precipitating factors [18,24,38], with higher rates of parental conflict reported by White males compared with South Asian males [16]. One study noted that disciplinary crises were common in both South Asian and White self harming adolescents, but that among South Asians this revolved around cultural issues [18]. Cultural conflict was associated with poisoning in 17 of 19 South Asian participants [18]; Biswas et al also noted culture conflict as a reason for self-harm in South Asian females rather than males [16]. Problems with parents, schoolwork, and boy or girl friends were more common amongst White adolescents, and problems with siblings were more common among South Asian adolescents [21].

There were few studies of discrimination. Goddard et al concluded that persecution, discrimination and migration related problems were more common among Black compared with White adolescents, especially in males [23]. Few studies examined service use, but in two studies, no ethnic differences were reported in follow-up or service usage [18,23,24]. Bhugra et al reported that South Asian adolescents who self harmed mostly did so impulsively and without planning [24]. In contrast Kingsbury et al, in a small study of adolescents, found longer premeditation times among South Asian compared with White adolescents [21].

#### Methods of Self Harm

Self-poisoning was the most common method reported among adolescents for all ethnic groups. Analgesics were the most commonly used substance [21,24,38]. However, Biswas et al noted that South Asian female adolescents were more likely to use "non-ingestants" (e.g household disinfectant, mouse poisons) than White adolescent girls [16]. In contrast, Handy et al's study of adolescent self-poisonings showed that there was little difference in the type of self-poisoning method used by young White and South Asian adolescent [18].

#### Comparative Rates

McGibben et al showed that although the rates for self poisoning for South Asian and White female adolescents did not differ significantly, both South Asian and White females were significantly more likely to be admitted for self poisoning than boys [13]. Only amongst females aged 10–14 were the rates of self harm higher among White compared with South Asian subjects [24]. The only school based study reported a lower risk of self harm among South Asian girls in the unadjusted analyses, but this finding was not sustained in multivariate analyses [19].

## Discussion

### Methodological Issues

Most studies could not be subjected to meta-analyses due to distinct outcomes, samples and methods; specifically methods of ethnic classification varied and changed over time making time trends difficult to discern. Only two studies could be pooled [14,17] offering further support for there being a higher risk of self harm among South Asian women. The inability to undertake meta-analyses on a larger number of studies reflected that publications did not include information on numerators and denominators, and census data were not always available for the study periods of interest or the age and gender group, for the local geographical areas in which the studies were conducted.

There is little good quality data comparing self harm in different ethnic minority groups in the UK. Most of the studies have significant methodological limitations, perhaps because these were the first to investigate self harm and ethnicity. Few explicitly presented numerator and denominator data, or used validated measures of mental disorder and deliberate self harm. Sample sizes were often small, and some studies did not set out to assess ethnic differences but reported these. Most of the studies used clinical samples rather than community samples, and none followed pathways into care. There were only two national studies; one investigated adult admissions for self poisoning in England, Scotland and Wales [25]; the other was a self report school based study of adolescents [19]. Differences in the findings across the studies may reflect variation of access to services as well as actual differences in rates and risk factors between ethnic minority populations.

### Demographic & Cultural Risks

The available evidence suggests that women of South Asian origin are over-represented in self harming samples compared to white women and men. There is some evidence that those of South Asian origin and aged under 35 are at higher risk than those over 35. There is also evidence of increasing risk in those of Caribbean origin aged less than 35 years [27,36]. However, there were few studies among adolescents, and the findings are inconsistent; one study showed a higher risk [24] and one a lower risk [19] of self harm among South Asian girls.

The most common method of self harm was self poisoning with analgesics or psychotropics. Previous histories of repeated self harm were reported less often in South Asian and Caribbean adults. South Asian and Caribbean origin people appeared to be less likely to show previous episodes of self harm, were less likely to have mental disorder in some studies, and were more likely to self harm impulsively. These findings will need to be considered by practitioners when assessing South Asian and Caribbean people for risk of self harm.

There have been some attempts to investigate possible reasons for different rates of self harm across ethnic groups. However, the results of these studies are not always consistent for South Asian and Caribbean origin people. Studies that report "culture conflict" in order to explain differential rates of suicide in South Asian and white groups are difficult to interpret. It was not always clear what was meant by culture conflict in the White group, and some attributions of culture conflict appeared to label social and interpersonal conflicts where these were judged by authors to be of cultural origin. There was rarely a validated instrument to assess cultural genesis of distress, for example, due to strain of gender roles, or of religious roles. It is unlikely that the questions asked to Asians about cultural practices, for example arranged marriages, would have had much significance or meaning in the white group, this perhaps explaining the low endorsement rates for these questions (0% and 1.8%).

One study demonstrated a link between ethnic minority density of population and risk of self harm [35]. Because explanatory factors such as social support and racism were not collected it is not clear what exactly is mediating the relationship between ethnic density and self harm rates. As only 50% of people who self harm come into contact with services, it is not clear whether the rates of self harm themselves are increased or whether the rates of presentation to services can explain some of the reported ethnic variations.

### Future Research

Better definition of ethnic groups is essential, especially for comparisons over time and internationally. Population studies following individuals into help seeking, from conventional and non-conventional sources of help may unravel the most appropriate care pathways for prevention among diverse ethnic groups. Of note there were no reports of self harm in refugee and asylum seekers or prison populations. Follow up studies using validated measures of mental distress and self harm are necessary for better prediction of risk for different ethnic groups. Population based studies that follow individuals through their care pathways are necessary in order to fully discern whether culturally determined patterns of help seeking or whether limited access to services account for the ethnic variations of rates in services. New patterns of immigration from Europe and the influence of globalisation on cultural practices also challenge conventional paradigms of researching ethnic inequalities.

## Conclusion

This review finds some ethnic differences in the nature and presentation of self harm. For example, compared to their white counterparts, Asian women were more likely to self harm and Asian men were less likely to self harm. The overall findings argue for ethnic specific preventive actions. However, the literature does not comprehensively cover the UK's diverse ethnic groups. Future prospective studies will need to consider new ethnicities.

## Competing interests

The author(s) declare that they have no competing interests.

## Authors' contributions

KB was the grant holder, KM was a co-PI and FR the research assistant employed on the grant. FR undertook searches and followed the methodology devised by KB and KM and strengthened in conjunction with the steering group. The manuscript was drafted first by FR, and then finalised by KB with comments on consecutive drafts by KM. All three authors took part in the analysis and tabulation of the data.

## Pre-publication history

The pre-publication history for this paper can be accessed here:



## Supplementary Material

Additional file 1 additional file y. Clinical Risk Factors & Methods for Self Harm in Ethnic Groups in the UK. Click here for file
