# Supplementary material for: Rates, risk factors & methods of self harm among minority ethnic groups in the UK: a systematic review
Source: BMC Public Health. 2007 Nov 19;7:336. doi: 10.1186/1471-2458-7-336 (PMC2211312; doi:10.1186/1471-2458-7-336)
Supplement: Additional file 1 — additional file y. Clinical Risk Factors & Methods for Self Harm in Ethnic Groups in the UK. [file 1471-2458-7-336-S1.doc]

**Additional File Y: Clinical Risk Factors & Methods for Self Harm in Ethnic Groups in the UK**

| **Author** | **Clinical Risk Factors for Self Harm** | **Methods of Self Harm** |
| --- | --- | --- |
|  | **Adult Studies** |  |
| Bagley et al 1972 | Situational stress diagnoses more common among Black group (48% vs 12%; R ^2^ =0.27, p<0.01)  Age: Black people younger than whites (mean 20.2 vs 27.2 years, p<0.01)  Being Black predicted repeat suicide in-patients who did not receive psychiatric treatment or social support at initial suicide attempt (r=0.28)  Discharge from hospital without treatment more common in Blacks (p<0.05) |  |
|  |  |  |
| Bhugra et al 2004 | Arguments with parents were most common factor (29%)  Alcohol problems at home very uncommon  Female adolescents were most likely to show adjustment problems irrespective of ethnicity  1/5 of white girls and ¼ of South Asian girls admitted to regular use of alcohol  South Asian males more likely to be playing truant  School problems in over 45% of subjects  South Asian females more likely to report cultural conflict  42% white females admitted to feeling depressed, compared with 37% white males, and 16% of South Asian males and females.  Logistic regression model showed culture conflict and low self esteem related to behavioural problems (overall p<0.05) | Overdoses most common methods:  45% paracetamol only; 14% paracetamol plus another substance  Impulsive in 42% and planned in 16% of self ham attempts  No South Asians reported regrets whereas 66% white females and 25% of white males expressed regret |
| Bhugra et al 1999a |  | 87.5% of attempters used poisonous substance: paracetamol or other painkiller most commonly used substance (40%) followed by benzodiazepines (10%) and antidepressants (7%).  No significant difference in methods used across sex or ethnic group. 86 repeat episodes made by 48 patients, 80% repeaters " white" most by overdose. |
| Bhugra et al 1999b | Self Harmers Controls p value  Psychiatric history 59% 3% 0.002  Physical disorder 24% 2.7% 0.001  Mean GHQ score 15.5 (s.e. 9.9) 6.4(1.7) 0.002  CIS score 23.2(10.8) 7.2(7.6) <0.001  Born in the UK 1/3 28% NS  Previous self harm 48% 3.7%  Arranged marriage  Good idea 30% 88% 0.003  Relationship with  a white person 22% 0 0.02  Keep in contact  with relatives abroad 74% 100% 0.01  Miscellaneous events more common among self harmers 0.006  Among those born in the UK, events and number of miscellaneous events (loss, separation, family arguments), events related to relationships with opposite sex and events related to education were more common (p=0.04, p=0.006, p=0.02 respectively)  Significant differences in White v South Asian Self Harmers:  62% v 82% wanted to die p<0.01  36% vs 62% expressed regret p not reported  38% v 22% took tablets p not reported  37.9 vs 21.7 mean number of tablets taken p=0.02  68% v 38% South Asian women more often p=0.009  owner occupiers  72% v 13% used alcohol in attempt p not reported  52% v 27% depressed p not reported  11% v 48% no psychiatric disorder p not reported  36% v 24% depressed on CIS-R p not reported  20% v 58% had some psych disorder p not reported  51% v 27% no psych disorder p=0.0003  17% v 43% health related event p=0.0008  4% v 22% life event related to opposite sex p=0.04. |  |
| Burke 1976a | Reasons for DSH: women men  Interpersonal dispute 68% 46% NS  Physical disorder 14% 25% NS  Psychiatric diagnosis in 33% (women, 8 men; depression most commonest)  10 patients had previously received care, and 3 patients made repeat attempts in follow up period of observation | Domestic substances 19  Inhaled coal gas 2  Swallowed house hold substances 4  Analgesics 1  Other tablets 2  South Asian men and women used similar methods : 64% swallowing psychotropic medication; 4% women and 29% men took alcohol at the time |
| Burke 1976b | None had previous DSH  None used alcohol  Substance taken  Psychotropics: 26%  Analgesics 25%  Dispute with lover (71%) or parent (7%) main reason.  Rates of attempted suicide higher compared to Caribbean natives but lower compared to British natives  Depression in 20 of 22 admissions  50/55 sent home whilst 5 transferred to psychiatric hospitals |  |
| Burke 1980 | Definitions of admission  Admission prone=type 2. At end of follow up, 21 patients readmitted.  The rest classified as Type 1.  Type 1 & 2 showed no ethnic patterning.  75% of attempted suicide among WI were type 1  83% of attempted suicides in Asians were type 1  Interpersonal disputes more common amongst type 1 than type 2 attempts (71% v 49%), p<0.05  Alcohol use more common among type 2 than type 1: 24% v 11%.  Marriage more common among Type 1 p<0.05  Numbers very small to offer strong conclusions |  |
| McKenzie et al 1995 | Black Caribbean White British difference (95%CI) p  Self harm over follow up period 4/43 (7.6%) 17/59 (28.8% ) -21.3% (-34.8 to -7.7) <.01  Antidepressants over follow up 7/53 (13.2%) 20/60 (33.3%) -20.1 (-35.1 to -5.1) <.01  Psychotherapy over follow up 1/53 (1.9%) 9/60 (15%) -13.1% (-22.9 to -2.4) <.01  Adjusted for class, age of onset, diagnosis, sex, length of follow up an catchment area  Risk of self harm over follow up: OR= 0.2, 95%CI: 0.1 to 0.7  Antidepressants over follow up: 0.3, 0.1 to 0.9  Psychotherapy over follow up: 0.2 0.01 to 1.6 |  |
| Merrill et al 1986 | Marital problems more common in South Asian women than White: 71.8% v 49.1%., p<0.001 (n= 85 (W), 225 (SA))  Fewer previous self harm attempts among South Asian women 34% vs 21.9%, p<0.01 (n=591(W) vs 146(SA))  Data Whites % (N) South Asians % (N)  Men  Culture Conflict: 0 (184) 11.1 (18) p<0.001  Personality Disorder 25.7(378) 8.2(49) p<0.025  Women:  No Psychiatric illness: 70.7 (392) 82.2 (135) <0.025  Alcoholism: 4.6(588) 0 (145) <0.025  Personality Disorder 22.1(588) 7.6(145) <0.0005  Culture Conflict 1.8 (272) 60.7 (56) <0.0005  Social Work Follow up 12.4(590) 24.7 (146) <0.0005  Asian women under 35yo less likely to have previous psychiatric treatment: 11.1% vs 20.1%, p<0.05 (n=393(W), 135(AS)) |  |
| Merrill et al 1987 | No ethnic differences on: marital problems, culture conflict (only in single females, mostly due to relationship with boys of a different colour)  West Indian % White %  Previous self poisoning Men 14.3 33.2 <0.05  Women 15.8 34 <0.001  Previous Psychiatric  Treatment Women <35 10.2 20.1 <0.05  Psychiatric Diagnosis Women <35 17.4 29.3 <0.05  Personality Disorder Women 6.3 22.1 <0.005  Alcoholism 1.5 6.6 <0.05  Psychiatric Social Men 14.3 3.7 <0.025  Worker follow up Women 28.4 12.4 <0.0005  Early separation from Father Women 23.2 12 <0.01  Mother Women 16.8 9.1 <0.005  Teenage pregnancy 9.1 1.8 <0.05 |  |
| Neeleman et al 2001 | Self harm rates vary by ethnic density in linear models comparing ethnic groups with White group.  Asian: RR=0.59 95%CI: 0.36-0.97  African Caribbean 0.76 0.64-0.9  Inverted U relationship between ethnic density and DSH in ethnic minorities, i.e. rates higher at low and high ethnic density.  Risk of DSH in whites also increased with ethnic density.  Significant findings only: Relative rates, 95%CI, per SD shift in ethnic density curve and adjusted for age, sex, area, and ethnic group.  White Ethnic minorities  Minority Density Area 1 1.79, 1.58-2.02, p<0.001 1.19, 1.01-1.41, p=0.039  AC density Area 1 1.84, 1.64-2.08, p<0.001 1.3, 1.09-1.53, p<0.003 i.e. increased risk with increasing AC density?  Area 2 0.71, 0.48-1.06, p=0.096  Asian density Areas1&2 0.85, 0.79-0.93, p<0.001 0.7, 0.54-0.9, p=0.006  Also differences of effect between areas one and two |  |
| Neeleman et al 1996 | DSH low in ethnic minorities, especially men.  DSH referral ratios for Indian women > than other women, 7.76(1.60-22.66).  More substance abuse in white group than Asians  Proportion of DSH patients who were unemployed: White: 39/83 (47%, 36-58) Ethnic minorities: 6/22 (27%,8-46)  W (n=83) EM(n=22)  Repeat DSH 69% 50% NS Psychiatric Diagnosis 77% 77% NS  Precipitant:  Psychosocial  Crisis 35% 59%  Physical Illness 5% 0  Substances Misuse 22% 5% diff 18% (4-30)  White: Unemployment associated with a 9.14 (5.02-16.62) increased DSH rate  Ethnic minorities: Unemployment associated with a 2.97 (1.16-7.60) increased DSH rate |  |
| Sheth et al 1994 | All Asian women migrated to UK after marriage, were housewives and married, had between 2-6 children  Burns from 25-99%  9/14 Asian women (64%) died  None had psychiatric history  Face, neck, trunk and upper limbs typical site of burns.  6 white patients who died all had psychiatric history  Little information on white Patients, not statistical comparisons | Most used paraffin to set fire |
| Wright 1981 | Cultural problems more common in Asians e.g. arranged marriages between people bought up in different cultures  Younger age in Caribbean and Asian people.  Greater increase in admission over 4 years for Caribbean people. .  C WI SA  Social factors: % % %  Previous Self poisoning 31 20 10  Separation fro parents 10 28 8  Precipitant:  Interpersonal problems 36 50 22  Cultural - - 28 | No significant differences in toxicological aspects of self poisoning between groups  Asians took fewer different drugs: 1.3 per patient episode, other groups 1.8/patient episode  Slight excess among Asians of Non-ingestants and anti-histamines (numbers too small for statistical comparison) |
|  | **Adolescent Studies** |  |
| Bhugra et al 2003 | No South Asians regretted attempt but 2/3 of white females did express regrets | Overdose most common method (60%)  15% of overdoses accompanied by self cutting  Nature of act planned by 16%  Impulsive 42% |
| Goddard et al 1996 | Males significantly under represented among DSH case: OR=0.14, 0.06-0.32  No differences between Black and White on: % females in groups, mean ages, rages of school absence, involvement in social services, family break up (<50% have both parents at home)  No ethnic differences on conduct disorders, emotional symptoms, or outcomes.  Persecution, discrimination & migration, social transplantation more common in the Black group compared to the White group (p<0.04).  These differences only significant in males. |  |
| Handy et al 1991 | Nuclear family: 21/25 Asians vs 6/25 Caucasians: p=0.01  Disrupted family: difference at p=0.01  Father employed: 11/22 Asians, 7/16 Caucasians, p=0.05  Cultural conflict as reason for poisoning more common in Asians 17/25 vs 0/25, p=0.1  Grief/loss more common in whites 4/25 vs 0/25, p<0.1  No differences in accidental, problems at school, disciplinary, relationship stress, physical abuse, experimentation: all very low numbers  Disciplinary crises common in both groups: Asian group revolved around cultural issues.  No further self harm: no ethnic differences in follow up findings. |  |
| Kingsbury 1994 | Long premeditation time in 46% Asians vs 11% of Caucasians, p=0.01  Friendship score lower in Asians, main questions on which differences apparent: friends near your house p=0.02, meet friends after school p=0.04, visit other friends’ houses 0.06.  Asians have lower frequency of seeing best friends, p=0.01  Trend: Asian children confide less in parents (NS), who are perceived as more controlling than Caucasians (p=0.01)  Problems more common among Caucasians: Parent % Schoolwork % boy/girlfriend % siblings% peers % numbers very small  Asian (n=13) 46% 38 23 31 23  Caucasian (n=39) 51% 49 57 8 24 | Analgesics used most commonly by Asians & Whites (69%, 56%) |
| Lockhart et al 1987 | 1983/4 cohort: WI patients more likely to be married/co-habiting p=0.006, unemployed p=0.05 |  |
| McGibben et al 1992 | In both groups girls significantly more likely to be admitted in hospital for Deliberate self poisoning |  |

ONS office of national statistics, OPCS office of population census & statistics, ICD International Classification of Diseases, W White, BA Black African, BC Black Caribbean, SA South Asian. WHO World Health Organisation, DSH Deliberate self harm, GHQ General Health Questionnaire, WI West Indian
